# Abnormal physiological findings after FFR-based revascularisation deferral are associated with worse prognosis in women

**DOI:** 10.1038/s41598-023-28146-6

**Published:** 2023-01-19

**Authors:** Masahiro Hoshino, Tim P. van de Hoef, Joo Myung Lee, Rikuta Hamaya, Yoshihisa Kanaji, Coen K. M. Boerhout, Guus A. de Waard, Ji-Hyun Jung, Seung Hun Lee, Hernan Mejia-Renteria, Mauro Echavarria-Pinto, Martijn Meuwissen, Hitoshi Matsuo, Maribel Madera-Cambero, Ashkan Eftekhari, Mohamed A. Effat, Koen Marques, Joon-Hyung Doh, Evald H. Christiansen, Rupak Banerjee, Chang-Wook Nam, Giampaolo Niccoli, Tadashi Murai, Masafumi Nakayama, Nobuhiro Tanaka, Eun-Seok Shin, Tetsuo Sasano, Yolande Appelman, Marcel Beijk, Paul Knaapen, Niels van Royen, Javier Escaned, Bon Kwon Koo, Jan J. Piek, Tsunekazu Kakuta

**Affiliations:** 1 Department of Cardiology, Tsuchiura Kyodo General Hospital, 4-1-1 Otsuno, Tsuchiura City, Ibaraki 300-0028 Japan; 2 Department of Cardiology, Amsterdam UMC – location AMC, Amsterdam, The Netherlands; 3 Department of Cardiology, Amsterdam UMC – location VUmc, Amsterdam, The Netherlands; 4 Department of Cardiology, NoordWest Ziekenhuisgroep, Alkmaar, The Netherlands; 5 Division of Cardiology, Department of Medicine, Samsung Medical Center, Heart Vascular Stroke Institute, Sungkyunkwan University School of Medicine, Seoul, Republic of Korea; 6 Division of Preventive Medicine, Department of Medicine, Brigham and Women’s Hospital and Harvard Medical School, Boston, MA USA; 7 Department of Epidemiology, Harvard T.H. Chan School of Public Health, Boston, MA USA; 8 Sejong General Hospital, Sejong Heart Institute, Bucheon, Korea; 9 Division of Cardiology, Department of Internal Medicine, Chonnam National University Hospital, Gwangju, Korea; 10 Hospital Clínico San Carlos, IDISSC, Universidad Complutense de Madrid, Madrid, Spain; 11 Hospital General ISSSTE Querétaro - Facultad de Medicina, Universidad Autónoma de Querétaro, Querétaro, México; 12 Department of Cardiology, Amphia Hospital, Breda, The Netherlands; 13 Department of Cardiovascular Medicine, Gifu Heart Center, Gifu, Japan; 14 Department of Cardiology, Tergooi Hospital, Blaricum, The Netherlands; 15 Department of Cardiology, Aarhus University Hospital, Aarhus, Denmark; 16 Division of Cardiovascular Health and Disease, University of Cincinnati, Cincinnati, OH USA; 17 Department of Medicine, Inje University Ilsan Paik Hospital, Goyang, South Korea; 18 Division of Cardiovascular Health and Diseases, Veteran Affairs Medical Center, University of Cincinnati Medical Center, Cincinnati, USA; 19 Department of Medicine, Keimyung University Dongsan Hospital, Daegu, South Korea; 20 Department of Cardiovascular Medicine, Institute of Cardiology, Catholic University of the Sacred Heart, Rome, Italy; 21 Cardiovascular Center, Yokosuka Kyosai Hospital, Yokosuka, Japan; 22 Cardiovascular Center, Toda Central General Hospital, Toda, Japan; 23 Department of Cardiology, Tokyo Medical University Hachioji Medical Center, Tokyo, Japan; 24 Department of Cardiology, Ulsan University Hospital, University of Ulsan College of Medicine, Ulsan, South Korea; 25 Department of Cardiovascular Medicine, Tokyo Medical and Dental University, Tokyo, Japan; 26 Department of Cardiology, Radboud University Medical Center, Nijmegen, The Netherlands; 27 Department of Internal Medicine, Cardiovascular Center, Seoul National University Hospital, Seoul, Republic of Korea

**Keywords:** Physiology, Cardiology

## Abstract

The prognostic value of abnormal resting Pd/Pa and coronary flow reserve (CFR) after fractional flow reserve (FFR)-guided revascularisation deferral according to sex remains unknown. From the ILIAS Registry composed of 20 hospitals globally from 7 countries, patients with deferred lesions following FFR assessment (FFR > 0.8) were included. (NCT 04485234) The primary clinical endpoint was target vessel failure (TVF) at 2-years follow-up. We included 1392 patients with 1759 vessels (n = 564 women, 31.9%). Although resting Pd/Pa was similar between the sexes (*p *= 0.116), women had lower CFR than men (2.5 [2.0–3.2] vs. 2.7 [2.1–3.5]; *p *= 0.004). During a 2-year follow-up period, TVF events occurred in 56 vessels (3.2%). The risk of 2-year TVF was significantly higher in women with low versus high resting Pd/Pa (HR: 9.79; *p *< 0.001), whereas this trend was not seen in men. (Sex: P-value for interaction = 0.022) Furthermore, resting Pd/Pa provided an incremental prognostic value for 2-year TVF over CFR assessment only in women. After FFR-based revascularisation deferral, low resting Pd/Pa is associated with higher risk of TVF in women, but not in men. The predictive value of Pd/Pa increases when stratified according to CFR values, with significantly high TVF rates in women in whom both indices are concordantly abnormal.

**Clinical Trial Registration:** Inclusive Invasive Physiological Assessment in Angina Syndromes Registry (ILIAS Registry), NCT04485234.

## Introduction

Previous studies investigating differences in fractional flow reserve (FFR) across sexes showed that stenoses of similar angiographic severity are less likely to generate ischemic FFR values in women when applying the uniform contemporary FFR threshold^1,2^. Some of these studies have found that, at a difference with FFR, instantaneous wave-free ratio (iFR) values are not influenced by sex^2^. As sex‐related differences may affect therapeutic decision-making, potential sex-related differences in the prognostic value of physiological testing in patients with intermediate coronary artery disease (CAD) is an important clinical concern.

The outlined sex differences in pressure-based coronary indices may have an origin in the microcirculation. Because FFR values have been reported to be influenced by the presence of coronary microvascular dysfunction (CMD), patients with FFR-based deferral and concomitant CMD may have a higher risk of long-term events than similar patients with a normal microcirculation. Yet, evidence on this topic is scarce and sometimes even paradoxical when analyzed from the sex perspective. In patients with FFR-based deferral of revascularisation it has previously been noted that, despite women showing lower values of coronary flow reserve (CFR) than men, long-term outcomes are similar between men and women^3^. Tentative explanations for the paradoxical findings outlined above include the presence of higher resting flow, smaller vessel size and smaller left ventricular mass in women, part of which impact FFR, but particularly changes in resting flow may also impact resting pressure measurements^3–5^.

On these grounds, we sought to investigate the relationship between patient sex, resting pressure indices and CFR, and the prognostic impact of this association in patients in whom coronary revascularization was deferred after FFR assessment.

## Method

### Study population

The ILIAS (Inclusive Invasive Physiological Assessment in Angina Syndromes) registry is a global, multi-center initiative pooling lesion-level coronary pressure and flow data, as well as vessel-level clinical outcome data. The registry is composed of 20 expert medical institutes from the Netherlands, Korea, Japan, Spain, Denmark, Italy and the United States of America. All data were prospectively gathered in local study protocols. Patients who underwent clinically indicated invasive coronary angiography and comprehensive invasive physiological assessment of at least one native coronary artery were enrolled in the registry. We enrolled patients who underwent clinically indicated invasive coronary angiography due to the reasons as follows: (1) symptoms suggestive of coronary artery disease, such as chest pain. (2) new or worsening chest symptoms. (3) abnormal results on a noninvasive stress imaging test. Patients with hemodynamic instability, significant valvular disease and prior coronary artery bypass graft surgery, as well as culprit vessels of acute coronary syndromes were excluded. Individual patient data for pooled analysis were collected using standardized spreadsheets and a fully compliant cloud-based clinical data platform (Castor EDC, Amsterdam, The Netherlands). Standardized definitions were used for all variables. ILIAS Registry was registered at Clinicaltrials.gov (ClinicalTrials.gov Identifier: NCT04485234).

From a total of N = 3046 vessels in the ILIAS Registry, patients with deferred lesions after FFR assessment were included. We excluded vessels with missing values in pre-PCI physiological indices including FFR, CFR, and resting Pd/Pa. We also excluded patients with acute ST-elevation myocardial infarction (MI) or missing age and sex, leaving N = 2100 vessels. Lesions with FFR ≤ 0.8 were excluded. Finally, 1759 vessels in 1392 patients with complete follow-up represented the study population in this analysis. (Supplemental Figure 1).

### Coronary angiography and physiological assessment

Coronary angiography and intracoronary testing were performed in all institutions using similar, standard techniques. After diagnostic coronary angiography, invasive physiological indices were measured using either separate pressure- (PressureWire, RADI medical – now Abbott Vascular, St Paul, MN ) and Doppler velocity sensor-equipped coronary guidewires (FloWire, Endosonics – now Philips-Volcano, San Diego, CA), dual pressure- and Doppler flow velocity-equipped guide wire (ComboWire, Volcano Corp. – now Philips-Volcano, San Diego, CA), or a temperature-sensitive pressure sensor-equipped guide wire (PressureWire, St Jude Medical- now Abbott Vascular, St. Paul, MN) using routine techniques. Intracoronary nitrate (100 or 200 μg) was administered before physiologic measurements. Using the Doppler velocity technique, baseline (bAPV) and hyperemic average peak flow velocities (hAPV) were labeled baseline and hyperemic flow, respectively. Using the coronary thermodilution technique, resting and hyperemic thermodilution curves were obtained in triplicate using three injections (4 mL each) of room‐temperature saline, and the inverse of the average basal (bTmn) and hyperemic mean transit times (hTmn) were labeled baseline and hyperemic flow, respectively. Hyperemia was induced by intravenous infusion of adenosine (140 μg/kg per min) or adenosine triphosphate (ATP) (150 μg/kg per min) through a peripheral or central vein, intracoronary bolus injection of adenosine (20-200mcg), or intracoronary bolus injection of nicorandil (3 mg), according to local standards. Resting Pd/Pa was calculated as the ratio of mean distal coronary pressure to mean aortic pressure during resting state, and resting Pd/Pa ≤ 0.92^6^was considered abnormal. CFR was calculated as the ratio of hyperemic to basal coronary flow, and CFR < 2.0^7^ was considered abnormal.

### Follow-up and clinical assessment

Clinical follow-up was obtained at outpatient clinic visits or by telephone contact to ascertain the occurrence of target vessel failure (TVF). TVF was defined as the composite of cardiac death, acute MI not clearly attributable to a nontarget vessel, and clinically driven revascularization of the target vessel by means of coronary artery bypass graft surgery or PCI. In the study, clinical endpoints were assessed by the 2-year incidence of TVF for the effect of sex on the prognostic impact of the resting coronary flow index in patients whose revascularization was deferred after measuring FFR. All patient-reported events were verified by evaluating hospital records or contacting the treating cardiologist or general-practitioner.

### Declarations section

This study was conducted in compliance with the guidelines of the Institutional Ethics Committee of Tsuchiura Kyodo General Hospital and received its approval (TKGH-IRB 2021FY98). This study also complied with the Declaration of Helsinki for investigation in human beings, and all patients provided written informed consent before enrollment.

### Statistical analysis

Data were analyzed on a per-patient basis for clinical characteristics and on a per-vessel basis for all other calculations. Continuous variables are presented as mean ± SD or median (first, third quartile [Q1, Q3]) and were compared with the Student *t*-test or Mann–Whitney U test. Categorical variables are presented as counts and percentages and were compared using Pearson’s chi-square test or Fisher exact test. Data including clinical outcomes were analyzed on a per-vessel basis. The cumulative incidence of 2-year TVF was presented as Kaplan–Meier curves and compared using a log-rank test. Event rates over time across groups defined by each sex and normal/abnormal physiological indices were visualized using the Kaplan–Meier method. To identify the independent predictors associated with 2-year TVF, multivariable Cox regression analysis was performed with covariates including diabetes, hyperlipidemia, CFR, and resting Pd/Pa. Three prediction models were constructed to determine the incremental discriminatory and reclassification performance of CFR and resting Pd/Pa for 2-year TVF. As a baseline, the clinical model 1 was derived from age, hypertension, diabetes mellitus, hyperlipidemia. The clinical model 2 was derived from clinical model 1 + CFR. In the clinical model 3, Pd/Pa was added. The discrimination ability of the models was estimated using the area under the receiver-operating characteristic curve (AUC) based on logistic regression, and the comparison of AUC between models was performed by using DeLong's method. Furthermore, the net reclassification index (NRI) and integrated discrimination improvement (IDI) were calculated to assess the reclassification performance.

All analyses were two-tailed, and statistical significance was defined as *P *< 0.05. Statistical analyses were performed using SPSS 25.0 for Windows (SPSS-PC, Chicago, IL, USA), and R version 3.6.0 (R Foundation for Statistical Computing, Vienna, Austria).

## Result

### Baseline characteristics

**Figure 1:**
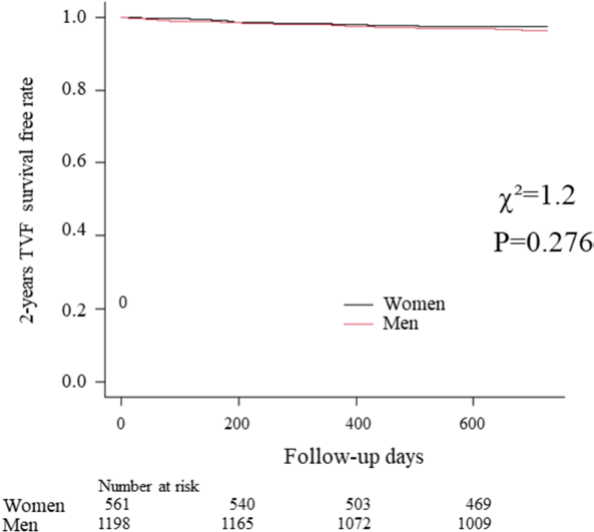
Kaplan–Meier time to event curves for target vessel failure during 2-year follow-up across the sexes. The 2-year TVF survival free rate was compared across the sex. *TVF* Target vessel failure.

Baseline characteristics of the study population are shown in Table 1. The mean (SD) age was 63.2 (10.1) years and there were 428 (30.8%) women in 1392 patients included in the present analysis. Clinical presentation was UAP or NSTE-ACS in 146 (10.5%) patients. Median (Q1, Q3) FFR, resting Pd/Pa, and CFR were 0.90 (0.86, 0.95), 0.97 (0.94, 0.99), and 2.6 (2.0, 3.4), respectively. Among 1759 vessels, 889 (50.5%) had an LAD lesion, with evidence of mild to intermediate epicardial disease (diameter stenosis: 44.8 ± 16.6%; FFR: 0.90 [Q1, Q3: 0.86, 0.95]). Compared with men, women had higher FFR (*p *< 0.001), lower CFR (*p *= 0.004), and higher Pd and Pa at rest and hyperemia. There was no significant sex difference in the resting Pd/Pa (*p *= 0.116). The median CFR was 2.7 (Q1, Q3: 2.1, 3.5) and 2.5 (Q1, Q3: 2.0, 3.2), and the median resting Pd/Pa was 0.97 (Q1, Q3: 0.94, 0.99) and 0.97 (Q1, Q3: 0.94, 0.97), for men and women, respectively. Whether using the Doppler velocity technique or the thermodilution method, resting coronary flow was not associated with resting Pd/Pa, regardless of sex. (Supplemental Figure 2) Supplemental Table 1 showed physiological findings in men and women in Pd/Pa ≤ 0.92 vs > 0.92. Physiological findings in men and women according to the events were shown in Supplemental Table 2 (Fig. 1).

**Table 1:** Baseline characteristics.

	Total (1392 patients)	Women (428 patients)	Men (964 patients)	*P* value
Age, y	63.2 ± 10.1	64.3 ± 10.5	62.7 ± 9.9	0.006
Hypertension	799 (57.4)	257 (60.0)	542 (56.5)	0.242
Diabetes mellitus	362 (26.0)	97 (22.7)	265 (27.6)	0.062
Hyperlipidemia	874 (62.8)	251 (58.6)	623 (64.8)	0.032
Current smoker	303 (21.8)	68 (16.0)	235 (24.8)	< 0.001
Previous MI	236 (17.0)	52 (12.2)	184 (19.1)	0.002
Clinical status (NSTEMI)	146 (10.5)	41 (9.6)	105 (10.9)	0.514
	(1759 vessels)	(561 vessels)	(1198 vessels)	
**QCA**				
Diameter stenosis	44.8 ± 16.6	43.6 ± 16.9	45.3 ± 16.5	0.076
Lesion length	10.3 (6.5–15.9)	10.0 (6.4–15.4)	10.5 (6.6–16.8)	0.460
Lesion location (LAD/RCA/LCx)	1228/418/113	309/130/116	580/312/297	0.025
**Physiological parameters**				
Resting Pd/Pa	0.97 (0.94–0.99)	0.97 (0.94–0.99)	0.97 (0.94–0.99)	0.212
Pd at rest	94 (84–94)	98 (87–109)	93 (84–103)	< 0.001
Pa at rest	98 (88–108)	100 (91–112)	96 (87–107)	< 0.001
FFR	0.90 (0.86–0.95)	0.92 (0.87–0.96)	0.90 (0.85–0.94)	< 0.001
Pd at hyper	81 (71–91)	85 (75–96)	79 (70–90)	< 0.001
Pa at hyper	90 (79–100)	93 (83–93)	88 (78–99)	< 0.001
CFR	2.6 (2.0–3.4)	2.5 (2.0–3.2)	2.6 (2.0–3.4)	0.007
Tmn at rest	0.69 (0.45–0.97)	0.60 (0.40–0.88)	0.74 (0.47–1.02)	< 0.001
Tmn at hyperemic	0.23 (0.17–0.32)	0.22 (0.16–0.31)	0.23 (0.17–0.32)	0.069
bAPV	15.7 (12.0–20.3)	16.3 (12.9–21.2)	15.3 (11.8–20.0)	0.007
hAPV	33.9 (30.2–49.2)	41.5 (33.1–50.4)	37.2 (29.0–48.4)	0.001

Data are presented as n (%) or median (Q1–Q3).

*MI* Myocardial infarction, *NSTEMI* Non-ST segment elevation myocardial infarction, *QCA* Quantitative coronary angiography, *LAD* Left anterior descending artery, *RCA* Right coronary artery, *LCx* Left circumflex artery, *FFR* Fractional flow reserve, *CFR* Coronary flow reserve, *Tmn* Mean transit time, *APV* Average peck flow velocity.

### Association of resting Pd/Pa and CFR with clinical outcomes

**Figure 2:**
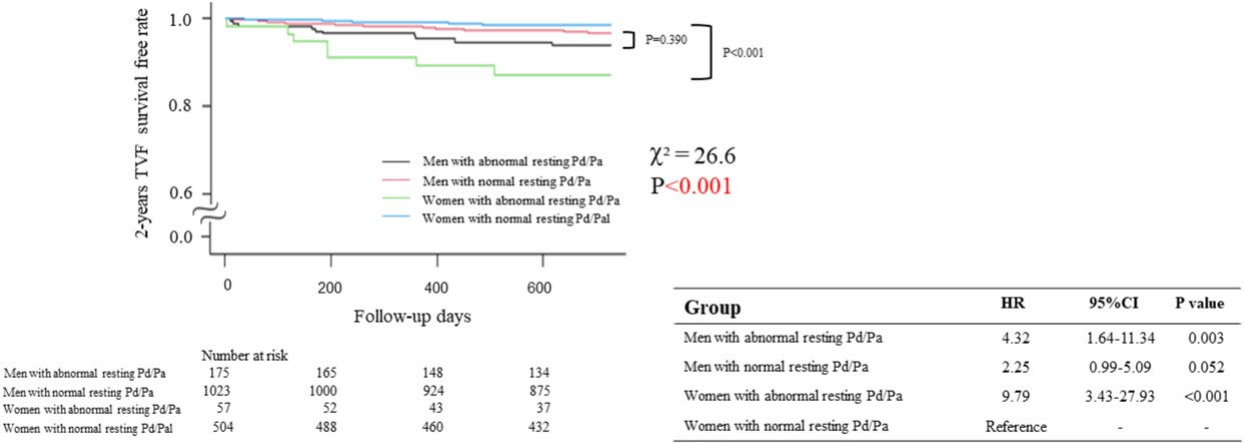
Kaplan–Meier time to event curves for target vessel failure during 2-year follow-up across the groups defined by normal/abnormal resting Pd/Pa and sex. The 2-year TVF survival free rate was stratified according to sex and resting Pd/Pa. In the risk comparison analyses, women with abnormal resting Pd/Pa showed the highest risk of 2-year TVF. Abbreviations are listed in Fig. 1.

A total of 56 TVF events (3.2%) were recorded during 2-year follow-up. There is no significant difference in the event rate or the results according to the vessel location involved and 2-year TVF. [Left anterior descending artery 26/897 (2.9%), right coronary artery 13/446 (2.9%), Left circumflex artery 17/416 (4.1%), *P *= 0.488.] Diabetes mellitus, low CFR, and resting Pd/Pa were found to be independent predictors of 2-year TVF in the overall population, (Table 2A) while in a subgroup analysis for each sex, resting Pd/Pa was identified as an independent predictor of TVF only in women. (Table 2B and 2C) Fig. 2 represents the cumulative incidence of TVF stratified according to sex and resting Pd/Pa during 2 years of follow-up. In the risk comparison analyses, women with abnormal resting Pd/Pa showed the highest risk of 2-year TVF. Women with abnormal resting Pd/Pa compared with normal one showed a hazard ratio of 9.79 (Fig. 2, 95% CI 3.43–27.93; *p *< 0.001). Seven women with abnormal resting Pd/Pa underwent 2-year TVF. (3 acute MI and 4 TVF).

**Table 2:** Univariate and multivariate cox regression analysis of predicting 2-year TVF.

	Univariable analysis			Multivariable analysis		
	HR	95% CI	*P* value	HR	95% CI	*P* value
**(A). Overall**						
Age	1.03	1.00–1.06	0.031			
Men	1.40	0.76–2.56	0.279			
Hypertension	1.08	0.63–1.83	0.789			
Diabetes mellitus	2.05	1.20–3.49	0.008	1.81	1.06–3.10	0.030
Hyperlipidemia	1.72	0.95–3.10	0.074	1.55	0.85–2.82	0.150
Diameter stenosis	1.03	1.01–1.05	0.002			
FFR	0.001	0.1 × 10^− 4^- 0.18	0.008			
Resting Pd/Pa (continuous variable)	0.2 × 10^− 4^	0.5 × 10^− 7^- 0.9 × 10^− 2^	< 0.001	0.1 × 10^− 3^	0.2 × 10^− 7^-0.08	0.006
CFR (continuous variable)	0.54	0.393–0.744	< 0.001	1.01	0.43–0.79	< 0.001
**(B). Women**						
Age	1.03	0.98–1.09	0.235			
Diabetes mellitus	1.38	0.43–4.39	0.588			
Hyperlipidemia	3.01	0.84–10.78	0.091			
Diameter stenosis	1.06	1.03–1.10	< 0.001			
FFR	3.4 × 10^− 11^	4.4 × 10^− 17 ^− 0.3 × 10^− 4^	< 0.001			
Resting Pd/Pa (continuous variable)	5.2 × 10^− 8^	6.6 × 10^− 11 ^− 0.4 × 10^− 4^	< 0.001	2.3 × 10^− 8^	9.5 × 10^− 12^–0.6 × 10^− 4^	< 0.001
CFR (continuous variable)	0.36	0.17–0.76	0.007	0.41	0.20–0.86	0.013
**(C). Men**						
Age	1.03	1.00–1.07	0.059	1.02	0.99–1.06	0.161
Diabetes mellitus	2.26	1.23–4.15	0.008	2.15	1.17–3.95	0.014
Hyperlipidemia	1.38	0.70–2.69	0.351			
Diameter stenosis	1.02	0.99–1.04	0.162			
FFR	0.17	0.7 × 10^− 3 ^− 42.5	0.533			
Resting Pd/Pa (continuous variable)	0.01	0.3 × 10^− 5 ^− 72.49	0.330			
CFR (continuous variable)	0.59	0.42–0.84	0.003	0.62	0.44–0.88	0.007

*TVF* Target vessel failure, *FFR* Fractional flow reserve, *CFR* Coronary flow reserve; *HR* Hazard ratio; *CI* Confidence interval.

**Figure 3:**
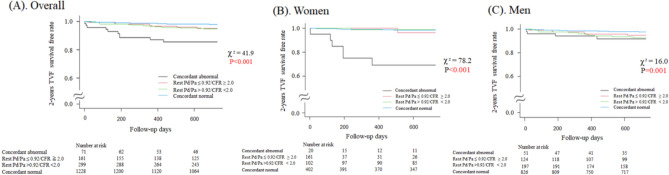
Kaplan–Meier time to event curves for target vessel failure during 2-year follow-up across the groups defined by normal/abnormal resting Pd/Pa and CFR. The 2-year TVF survival free rate was compared across the groups defined by normal/abnormal resting Pd/Pa and CFR in (**A**) overall cohort, (**B**) women, and **(C**) men. *CFR* Coronary flow reserve. Other abbreviations are listed in Fig. 1.

Figure 3A depicts the Kaplan–Meier curves for 2-year TVF across the groups defined by normal and abnormal resting Pd/Pa and CFR in patients in whom revascularization was deferred following FFR measurements. Concordant abnormal resting Pd/Pa and CFR group carried the highest risk for TVF, while in a subgroup analysis for each sex, these two indices were identified as independent predictors of 2-year TVF only for women. (Fig. 3B and 3C) These results were consistent with per-patient analyses. (Supplemental Figure 3 and 4.)

**Figure 4:**
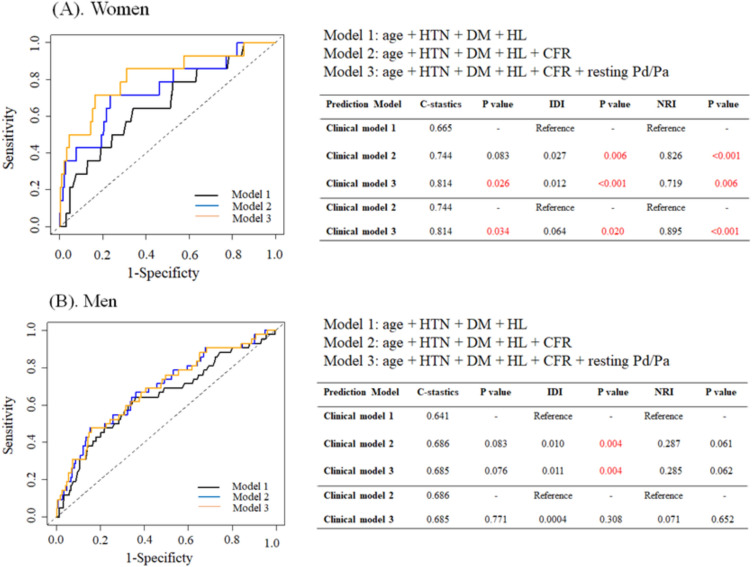
Comparison of ROC curves for clinical models to predict 2-year target vessel failure. The AUC of the ROC curve was improved by combining CFR and resting Pd/Pa in (**A**) women, whereas this trend was not seen in (**B**) men. *ROC* Receiver-operating characteristic, *AUC* Area under the curve. Other abbreviations are listed in Fig. 1 and Fig. 3.

ROC analyses also revealed differences between the sexes in the prognostic values of these measures. In women, compared with the clinical model 1 (AUC: 0.665), the model accuracy for 2-year TVF tended to increase with the addition of CFR (model 2, AUC: 0.744; *p *= 0.083). The model accuracy was further improved when resting Pd/Pa was added to the model 2 with the highest AUC (model 3, AUC: 0.814; *p *= 0.026), (Fig. 4A) whereas these trends were not seen in men. (Fig. 4B) Furthermore, the model 3 showed significant incremental reclassification ability for 2-year TVF (NRI: 0.895, *p *< 0.001; IDI: 0.064, *p *= 0.020) compared with the model 2 in women, whereas the model 3 provided no incremental reclassification ability for 2-year TVF in men (NRI: 0.071, *p *= 0.652; IDI: 0.004, *p *= 0.308).

The exploratory subgroup analyses indicated that resting Pd/Pa showed the quantitative interaction of the sex effect. No other significant interactions were observed. (Supplemental Table 3).

## Discussion

The aim of this study was to investigate, from a patient´s sex perspective, the prevalence and prognostic implications of abnormal physiological indices in patients with deferred coronary revascularization based on FFR. The key findings were that, compared with men, 1) women had higher resting coronary flow, and that 2) abnormal resting Pd/Pa was associated with an increased rate of 2-year TVF, particularly in women in whom CFR was also abnormal. Our study also confirmed the existence of an overall relationship between impaired CFR and mid-term outcomes after FFR-based deferral of revascularisation. The implications of these findings are discussed in the following paragraphs.

### Difference in physiological indices and prognosis between women and men

In this study, women had higher FFR, lower CFR, and similar resting Pd/Pa compared with men, and overall demonstrated a similar incidence of adverse cardiac events. (Fig. 1) The associations between CFR, resting coronary flow and sex noted in our study were previously reported by Wieneke et al.^8^ Women generally have older age, smaller vessel size^4^, higher resting coronary flow^3,9^, and a higher prevalence of microvascular dysfunction at the time of physiological assessment^10^, all of which may impact coronary physiological indices differences between the sexes.

Compared with FFR, there have been limited reports on the sex differences in resting coronary pressure indices. In accordance with previous studies^2,11^, there were no significant differences in resting Pa/Pa values between the sexes in the current analysis, and the angiographic stenosis severity was also similar.

Previously, despite a resulting lower revascularization rate in women, iFR- and FFR-guided strategies showed comparable clinical outcomes, regardless of sex^2^. However, differences in prognostic value of resting Pd/Pa according to sex has not been well defined.

From a historical perspective, high resting flow was previously considered an innocent cause of false positive readings of CFR and, overall, a caveat of the index that should be taken into account in formulating alternative, corrected versions of CFR^8^. However, high resting coronary flow may be an epiphenomenon of physiological derangements at a systemic^12^ or cardiac level^13,14^, suggesting the presence of disturbed regulation of resting myocardial flow, and implicating a potential prognostic relevance. Studies on coronary flow responses to exercise have shown that the presence of high resting coronary flow with preserved maximal arteriolar vasodilation is associated to increased release of NT-proBNP, lower exercise coronary perfusion efficiency and higher rates of inducible myocardial ischaemia^15^. Speculative explanation can be done from this perspective that the prognostic value of abnormal CFR indicates not only an impaired maximal microvascular vasodilation of the coronary circulation, but also an abnormally high vasodilation at rest.

As previously described, women are less likely to experience a cardiovascular event than men, and furthermore, women remain significantly less likely to undergo revascularization than men^3,16,17^. Although the combined measurement of resting Pd/Pa and CFR has not been reported to identify specific coronary pathology underlying intermediate CAD, we hypothesized sex differences of resting coronary flow components could potentially enhance risk stratification, although no specific physiological mechanism has been determined.

### Impact of microvascular disease on sex differences

The WISE (Women's Ischemia Syndrome Evaluation) study^10^ showed that women with angina without obstructive coronary artery disease are more likely to have microvascular dysfunction assessed by CFR. However, a recent study on sex differences with invasive measurements of microvascular function showed that the hyperemic coronary flow and IMR were not different according to sex^3^. In contrast, resting coronary flow was noted to be higher in women, thereby potentially accounting for low CFR values^3^. Previous studies showed that small arteries with high resting coronary flow in women may provide a protective effect through the high endothelial shear stress, leading to a lower cardiovascular event rate^18,19^. The exact relationship between microvascular dysfunction in women with ischemia without significant epicardial stenosis and worse prognosis has not been completely elucidated. For understanding the specific coronary hemodynamics in the individual perfusion mechanisms, especially in women, information from combined assessment of FFR and CFR may not be sufficient, and our results strongly suggest the need for the assessment of resting coronary flow for the prognostic information, particularly in lesions of women with revascularization deferral by FFR > 0.80. Therefore, when we risk stratify patients with CAD, this study indicates the importance to consider the difference in prognostic values of both CFR and resting coronary flow or the combination of these two metrics between the sexes. Our data suggested that combined assessment of resting Pd/Pa and CFR, particularly in women, could help further risk stratification for patients with deferred revascularization after FFR assessment.

### Limitations

The results of this study should be interpreted with considering several limitations. First, this study is a subgroup analysis of ILIAS Registry (NCT 04,485,234) and enrolled patients with deferred lesions following FFR assessment (FFR > 0.8) in the real-world clinical practice. The current registry included younger patients, patients with lower prevalence of diabetes mellitus and hypertension and showed a small number of events during the follow-up period. However, the event rate was comparable to the previously published similar registry study^20^. This limitation precluded extensive subgroup analyses to explore the effect of sex on the prognosis. In addition, the registry included a relatively small number of events in women, which may not allow extensive subgroup analyses to determine the effect of sex on the prognostic information of the resting pressure index. Second, revascularization decision makings and subsequent treatment strategies were based on the operator’s discretion without the prospectively defined procedure algorithm. Although the present analysis was performed according to clinical guidelines applicable at the time of the coronary angiography, this may have resulted in a certain level of selection bias. Third, since this study analyzed the data from the international multicenter registry, the detailed records of medical management were not available in the present registry. Fourth, the enrolled patients had a symptom suggestive of angina or were considered indicative of invasive coronary angiograms, but the details of the severity of angina were not reported. Fifth, there were numerical differences in the number of registered patients and the event rates across the centers. The exact reason for this heterogeneity remains to be determined. Sixth, invasive physiological indices were obtained using different techniques by either Doppler velocity sensor-equipped coronary guidewires or temperature-sensitive pressure sensor-equipped guidewires in the present study, the poor-quality data acquisition with Doppler observed in approximately 30%^21^ of patients may cause some degree of heterogeneity or bias among the study populations from each center. Seventh, the incidence of TVF was based on physician reporting and was not adjudicated by the events committee. Finally, ILIAS Registry lacks measurements of cardiac mass and vessel size, which may be largely associated with the sex and physiological indices.

## Conclusions

In a large global registry, we found sex-related differences in the prognostic impact of resting coronary flow using resting Pd/Pa in patients with deferred revascularization based on FFR measurements. Women had higher FFR, lower CFR, and similar resting Pd/Pa compared with men, and showed similar cardiac events in the present large registry. However, in women in whom PCI was deferred after FFR evaluation, resting Pd/Pa provided a significant and incremental prognostic on top of CFR assessment in predicting future vessel-oriented adverse outcomes, which was not seen in men.

## Supplementary Information

Supplementary Information 1.

Supplementary Information 2.

Supplementary Information 3.

Supplementary Information 4.

## Data Availability

The datasets used and/or analyzed during the current study available from the corresponding author on reasonable request.

## Abbreviations

CI: Confidence interval
CAD: Coronary artery disease
CFR: Coronary flow reserve
CMD: Coronary microvascular dysfunction
FFR: Fractional flow reserve
HR: Hazard ratio
MI: Myocardial infarction
TVF: Target vessel failure
PCI: Percutaneous coronary intervention
