# Supplementary material for: Abnormal physiological findings after FFR-based revascularisation deferral are associated with worse prognosis in women
Source: Sci Rep. 2023 Jan 19;13:1027. doi: 10.1038/s41598-023-28146-6 (PMC9852478; doi:10.1038/s41598-023-28146-6)
Supplement: Supplementary file 1 — Supplementary Information 1. [file 41598_2023_28146_MOESM1_ESM.docx]

Supplemental Table 1. Physiological indices in women and men in resting Pd/Pa ≤ 0.92 vs > 0.92.

|  | **Women with resting Pd/Pa ≤ 0.92**  **(57 vessels)** | | **Women with resting Pd/Pa > 0.92**  **(504 vessels)** | **P value** | **Men with resting Pd/Pa ≤ 0.92**  **(175 vessels)** | **Men with resting Pd/Pa > 0.92**  **(1023 vessels)** | **P value** |
| --- | --- | --- | --- | --- | --- | --- | --- |
| **Resting Pd/Pa** | **0.91 (0.89-0.92)** | **0.98 (0.95-0.99)** | |  | **0.91 (0.90-0.92)** | **0.97 (0.95-0.99)** | **<0.001** |
| Resting Pd | 88 (80-96) | 99 (88-111) | | <0.001 | 85 (74-94) | 94 (85-104) | <0.001 |
| Resting Pa | 98 (90-107) | 101 (91-112) | | 0.112 | 93 (82-103) | 96 (87-107) | 0.001 |
| **FFR** | **0.85 (0.83-0.86)** | **0.92 (0.87-0.97)** | |  | **0.84 (0.82-0.86)** | **0.91 (0.86-0.95)** | **<0.001** |
| hyperemic Pd | 80 (68-85) | 86 (76-96) | | <0.001 | 74 (64-80) | 80 (71-90) | <0.001 |
| Hyperemic Pa | 90 (81-100) | 93 (83-103) | | 0.309 | 87 (74-96) | 88 (79-99) | 0.059 |
| **CFR** | 2.2 (1.5-2.6) | 2.6 (2.1-3.3) | | <0.001 | 2.6 (1.9-3.5) | 2.7 (2.1-3.5) | 0.155 |
| Resting Tmn | 0.44 (0.35-0.66) | 0.60 (0.42-0.90) | | 0.030 | 0.67 (0.46-1.00) | 0.74 (0.47-1.03) | 0.353 |
| hyperemic Tmn | 0.25 (0.17-0.29) | 0.21 (0.16-0.31) | | 0.310 | 0.24 (0.18-0.34) | 0.23 (0.17-0.32) | 0.342 |
| bAPV | 17.7 (14.4-23.1) | 16.2 (12.8-21.0) | | 0.210 | 16.0 (12.6-19.4) | 15.2 (11.7-20.0) | 0.786 |
| hAPV | 40.5 (30.0-44.8) | 41.5 (33.6-50.7) | | 0.471 | 34.7 (27.9-43.8) | 37.4 (29.4-49.0) | 0.132 |

Data are presented as n (%) or median (Q1-Q3).

FFR = fractional flow reserve, CFR = coronary flow reserve, Tmn = mean transit time, bAPV = basal average peak velocity, hAPV = hyperemic average peak velocity.
