# Supplementary material for: Abnormal physiological findings after FFR-based revascularisation deferral are associated with worse prognosis in women
Source: Sci Rep. 2023 Jan 19;13:1027. doi: 10.1038/s41598-023-28146-6 (PMC9852478; doi:10.1038/s41598-023-28146-6)
Supplement: Supplementary file 2 — Supplementary Information 2. [file 41598_2023_28146_MOESM2_ESM.docx]

Supplemental Table 2. Physiological indices in women and men according to the events.

|  | Women with events  (14 vessels) | | Women without events  (547 vessels) | P value | Men with events  (42 vessels) | Men without events  (1156 vessels) | P value |
| --- | --- | --- | --- | --- | --- | --- | --- |
| **Resting Pd/Pa** | 0.93 (0.91-0.95) | 0.97 (0.94-0.99) | | <0.001 | 0.96 (0.93-0.99) | 0.97 (0.94-0.99) | 0.296 |
| Resting Pd | 91 (80-107) | 98 (87-110) | | 0.244 | 89 (79-100) | 93 (84-103) | 0.072 |
| Resting Pa | 99 (93-115) | 100 (91-112) | | 0.869 | 95 (82-103) | 96 (87-107) | 0.117 |
| **FFR** | 0.85 (0.83-0.86) | 0.91 (0.87-0.96) | | <0.001 | 0.89 (0.85-0.94) | 0.89 (0.85-0.94) | 0.627 |
| hyperemic Pd | 79 (64-84) | 85 (75-96) | | 0.067 | 79 (67-87) | 79 (70-90) | 0.302 |
| Hyperemic Pa | 95 (79-100) | 93 (83-103) | | 0.824 | 88 (78-96) | 88 (78-99) | 0.421 |
| **CFR** | 2.0 (1.5-2.2) | 2.5 (2.0-3.2) | | 0.002 | 2.1 (1.7-3.1) | 2.7 (2.1-3.5) | 0.003 |
| Resting Tmn | 0.36 (0.33-0.51) | 0.60 (0.40-0.88) | | 0.259 | 0.65 (0.39-0.90) | 0.74 (0.47-1.03) | 0.340 |
| hyperemic Tmn | 0.29 (0.16-0.29) | 0.22 (0.16-0.31) | | 0.910 | 0.31 (0.20-0.45) | 0.23 (0.17-0.32) | 0.093 |
| bAPV | 19.8 (15.0-24.0) | 16.3 (12.8-21.0) | | 0.256 | 15.8 (12.4-22.3) | 15.3 (11.8-19.9) | 0.539 |
| hAPV | 37.0 (23.0-45.0) | 41.5 (33.4-50.5) | | 0.467 | 43.9 (28.0-51.9) | 37.1 (29.3-48.2) | 0.715 |

Data are presented as n (%) or median (Q1-Q3).

FFR = fractional flow reserve, CFR = coronary flow reserve, Tmn = mean transit time, bAPV = basal average peak velocity, hAPV = hyperemic average peak velocity.
