# Supplementary material for: Abnormal physiological findings after FFR-based revascularisation deferral are associated with worse prognosis in women
Source: Sci Rep. 2023 Jan 19;13:1027. doi: 10.1038/s41598-023-28146-6 (PMC9852478; doi:10.1038/s41598-023-28146-6)
Supplement: Supplementary file 3 — Supplementary Information 3. [file 41598_2023_28146_MOESM3_ESM.docx]

Supplemental Table 3. Exploratory subgroup analysis for TVF at 2-years according to the sex

|  | HR | 95% CI | P value | P for interaction |
| --- | --- | --- | --- | --- |
| Age | 1.03 | 1.00-1.06 | 0.031 |  |
| Women | 1.03 | 0.98-1.09 | 0.235 | 0.957 |
| Men | 1.03 | 1.00-1.07 | 0.059 |  |
| Diabetes mellitus | 2.05 | 1.20-3.49 | 0.008 |  |
| Women | 1.38 | 0.43-4.39 | 0.588 | 0.457 |
| Men | 2.26 | 1.23-4.15 | 0.008 |  |
| Hyperlipidemia | 1.72 | 0.95-3.10 | 0.074 |  |
| Women | 3.01 | 0.84-10.78 | 0.091 | 0.291 |
| Men | 1.38 | 0.70-2.69 | 0.351 |  |
| CFR | 0.541 | 0.393-0.744 | <0.001 |  |
| Women | 0.355 | 0.167-0.755 | 0.007 | 0.223 |
| Men | 0.592 | 0.418-0.837 | 0.003 |  |
| Resting Pd/Pa | 0.2×10⁻⁴ | 0.5×10⁻⁷- 0.9×10⁻^3^ | <0.001 |  |
| Women | 0.5×10⁻⁷ | 6.6×10⁻^11^ - 0.4×10⁻^4^ | <0.001 | 0.022 |
| Men | 0.015 | 0.3×10⁻^5^-72.5 | 0.330 |  |

Abbreviations: TVF = target vessel failure, CFR = coronary flow reserve; HR = hazard ratio; CI = confidence interval.
