# Supplementary material for: Abnormal physiological findings after FFR-based revascularisation deferral are associated with worse prognosis in women
Source: Sci Rep. 2023 Jan 19;13:1027. doi: 10.1038/s41598-023-28146-6 (PMC9852478; doi:10.1038/s41598-023-28146-6)
Supplement: Supplementary file 4 — Supplementary Information 4. [file 41598_2023_28146_MOESM4_ESM.docx]

**Supplemental material**

**Figure legends**

**Supplemental Figure 1. Study flow chart**

**
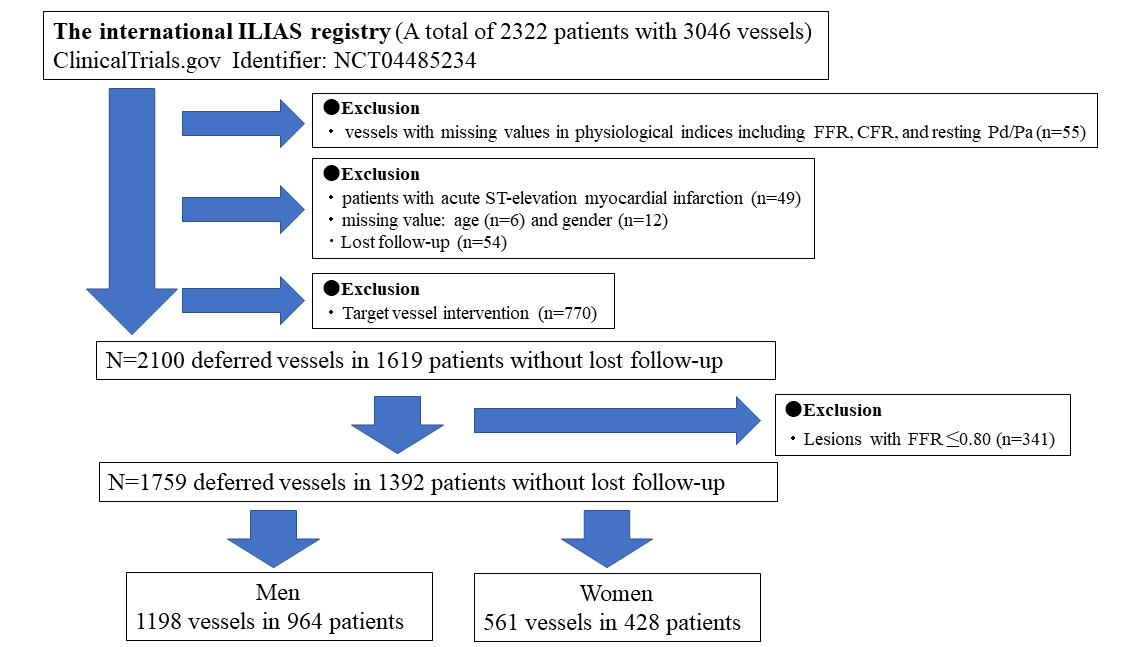
**

Abbreviations: FFR, fractional flow reserve, CFR, coronary flow reserve.

**Supplemental Figure 2. Association between resting Pd/Pa and resting flow component**

**
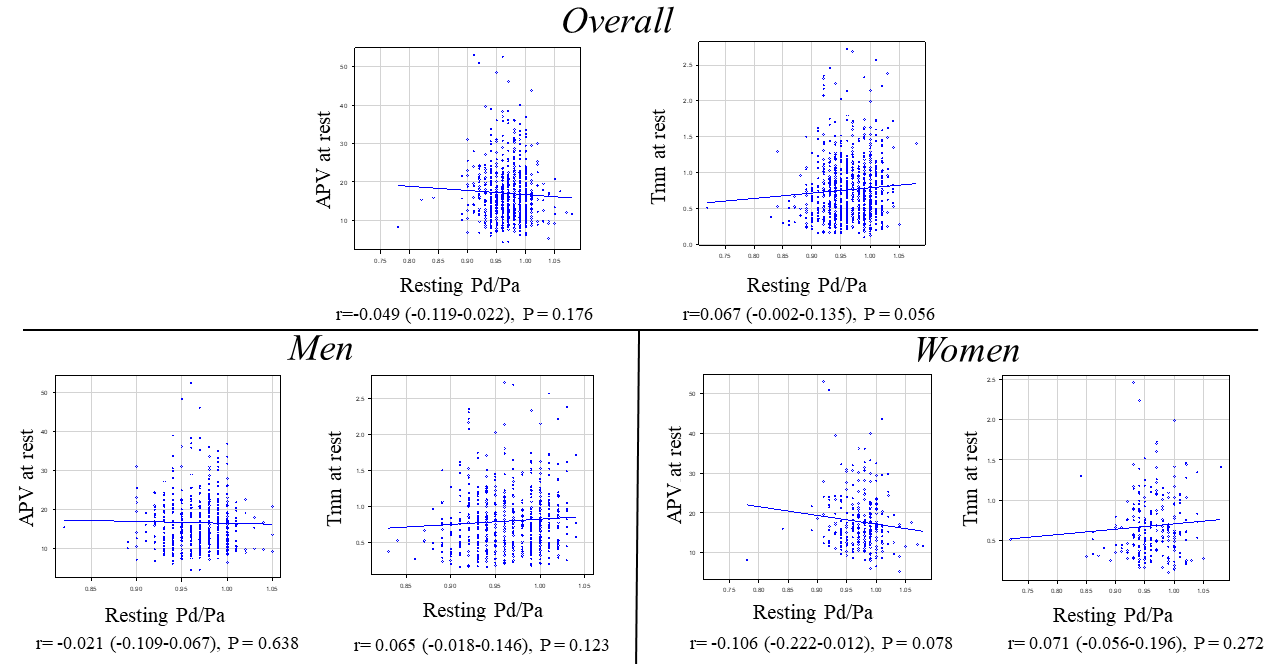
**

Resting coronary flow was not associated with resting Pd/Pa, regardless of sex.

Abbreviations: APV, average peak flow velocity, Tmn, mean transit time.

**Supplemental Figure 3. Kaplan–Meier analysis for target vessel failure during 2-year follow-up across the groups defined by normal/abnormal resting Pd/Pa and sex (Per-patient analysis)**

**
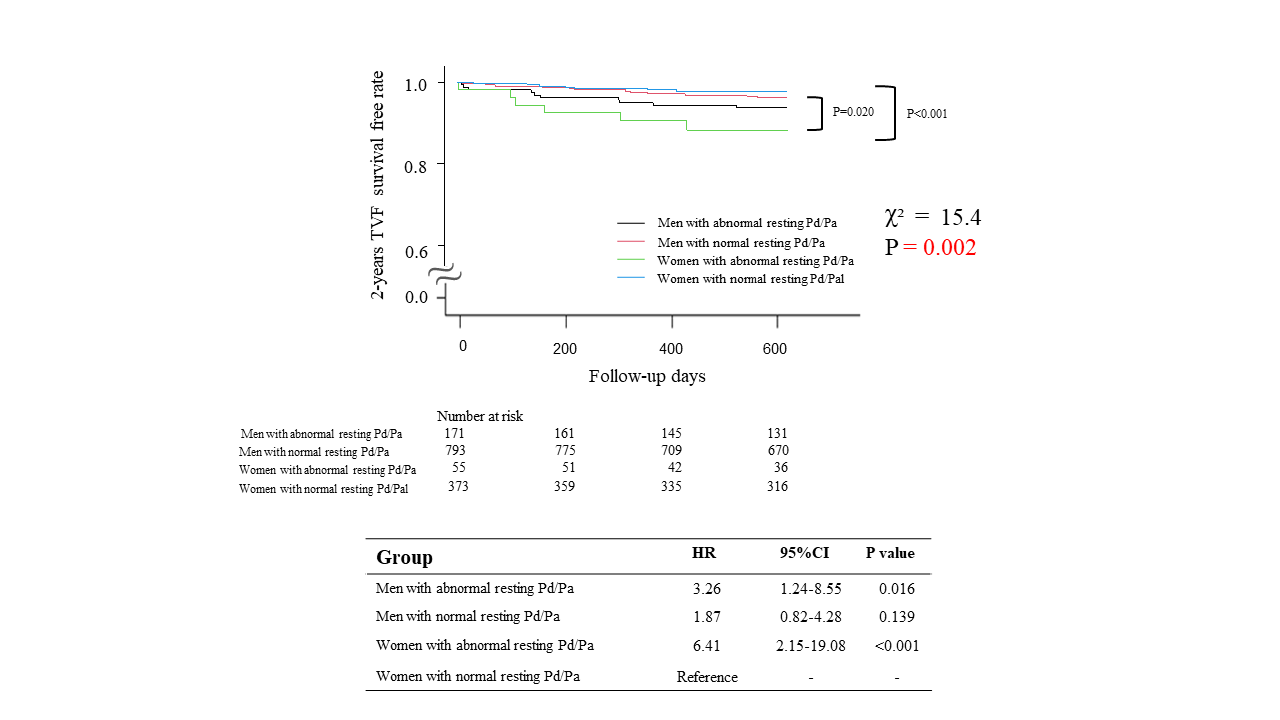
**

The 2-year TVF survival free rate was stratified according to sex and resting Pd/Pa. (Per-patient analysis)

In the risk comparison analyses, women with abnormal resting Pd/Pa showed the highest risk of 2-year TVF.

Abbreviations: TVF, target vessel failure.

**Supplemental Figure 4. Kaplan–Meier time to event curves for target vessel failure during 2-year follow-up across the groups defined by normal/abnormal resting Pd/Pa and CFR (Per-patient analysis).**

**
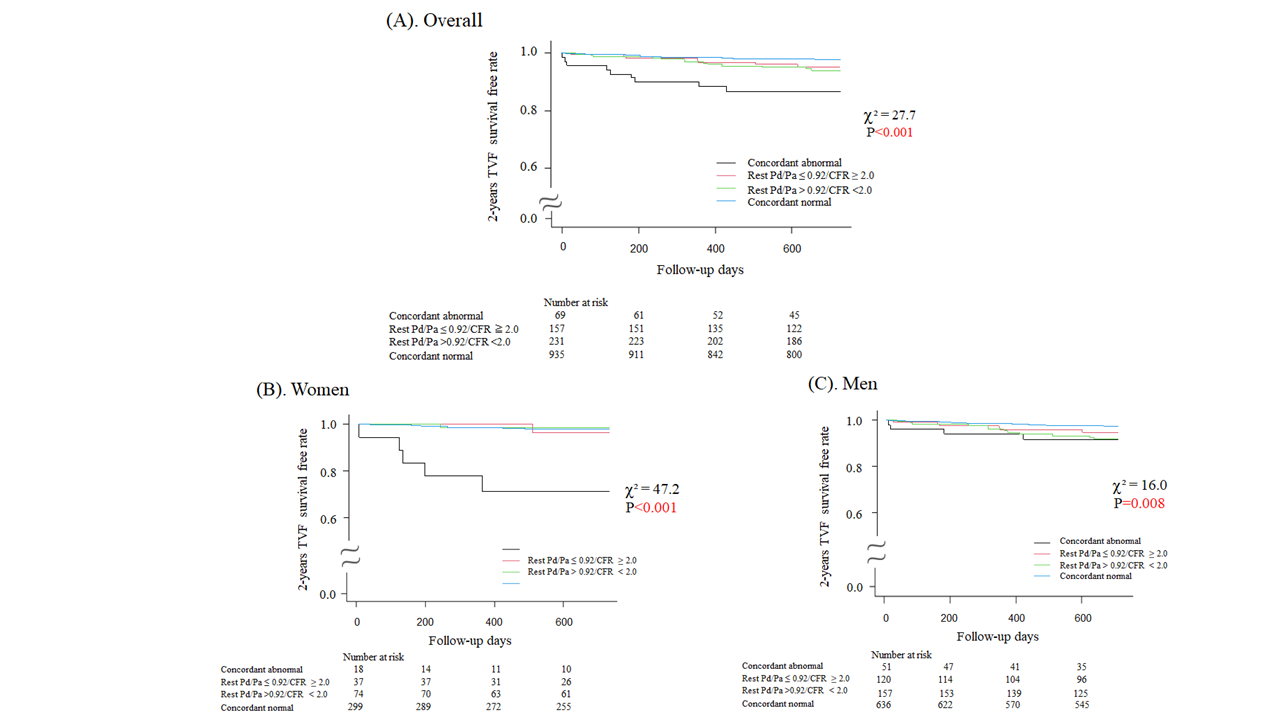
**

The 2-year TVF survival free rate was compared across the groups defined by normal/abnormal resting Pd/Pa and CFR in (A) overall cohort, (B) women, and (C) men. (Per-patient analysis)

Abbreviations: CFR, coronary flow reserve, TVF, target vessel failure.
